# Safety of Continuing Anticoagulation Prior to Cardiac Catheterization in Pediatric Patients: A Los Angeles Center Experience

**DOI:** 10.1007/s00246-023-03097-x

**Published:** 2023-02-02

**Authors:** Mounica Y. Rao, Patrick M. Sullivan, Cheryl Takao, Sarah Badran, Neil D. Patel

**Affiliations:** 1 Division of Pediatric Cardiology, Children’s Hospital Los Angeles, Keck School of Medicine, University of Southern California, Los Angeles, CA USA; 2 Congenital Heart Center, Spectrum Health Helen DeVos Children’s Hospital, Michigan State University College of Human Medicine, Grand Rapids, MI USA; 3 Division of Cardiology, Children’s Hospital Los Angeles, 4650 Sunset Blvd., MS 34, Los Angeles, CA USA

## Abstract

This is the first report specifically describing outcomes of pediatric patients who underwent cardiac catheterization while on uninterrupted anticoagulation. One hundred forty-four cardiac catheterizations were identified that met inclusion criteria at our institution from 11/2014 to 10/2019. The median age and weight of the patients were 6.3 [0.01–20.9] years and 19.3 [2.1–172.5] kg, respectively. Seventy-eight (54%) catheterizations involved inpatients. The most common cardiac diagnoses among the cohort were single ventricle (*n* = 41), conotruncal defects (*n* = 37), and structurally normal heart (*n* = 16). The most common indications for anticoagulation were arterial/venous thrombus (*n* = 45), Fontan physiology (*n* = 32), and mechanical valve thrombus prophylaxis (*n* = 27). The anticoagulation medications used were warfarin (*n* = 57), heparin (*n* = 52), enoxaparin (*n* = 25), fondaparinux (*n* = 5), rivaroxaban (*n* = 2), and both heparin and warfarin (*n* = 3). Interventions were performed in 96 cases (67%). The median length of the procedure was 122.5 [15–760] minutes, and the median time to achieve hemostasis was 18.0 [range: 5–76, IQR: 13–25] minutes. Adverse events were present in 11 cases (7.6%), and of those only 2 cases (1.4%) were bleeding-related complications. Our single-center data suggest that performing cardiac catheterization on pediatric patients while on uninterrupted anticoagulation is safe and does not substantially increase the risk of bleeding complications based on a cohort of patients that varied in age, size, diagnosis, medical complexity, and type of intervention performed. Patients on warfarin therapy for a mechanical valve are most likely to benefit from this practice, as the ability to continue warfarin therapy avoids the need for bridging and other interruption-related complications.

## Introduction

Anticoagulation is often held in pediatric patients undergoing cardiac catheterization based on adult guidelines and practices. In adults, oral anticoagulation agents are held (with or without heparin bridging) prior to cardiac catheterization due to concerns for potential bleeding complications [1, 2]. For femoral access, a recent consensus statement on best practices in the cardiac catheterization laboratory recommends holding warfarin until the international normalized ratio (INR) falls below 1.8 and holding direct oral anticoagulant (DOAC) therapy for a minimum of 24–48 h based on the operator’s perception of bleeding risk [3]. However, evidence in adults has shown that cardiac catheterization-related bleeding risk is similar in uninterrupted vitamin K antagonist (VKA) therapy compared to interrupted therapy, and uninterrupted VKA therapy has a lower risk of bleeding compared to interrupted VKA therapy with heparin bridging [4–6]. Similar findings have been demonstrated regarding hematoma formation between uninterrupted versus interrupted DOAC therapy prior to pacemaker or defibrillator implantation and prior to catheter ablation in atrial fibrillation [7–9].

In children undergoing cardiac catheterization, the incidence of bleeding or hematoma has been shown to be low at 0.6–0.8% [10, 11]. However, studies regarding the safety of performing a cardiac catheterization while on systemic anticoagulation are lacking. A survey conducted in 2018 demonstrated significant variation in preoperative antiplatelet agent and intraoperative anticoagulation practices among providers performing cardiac catheterization in children and adults with congenital heart disease [12]. Although the study only assessed practices regarding aspirin, clopidogrel, and intraprocedural heparin, it is likely that variability also exists in pre-procedural anticoagulation practices involving other systemic anticoagulation agents. At our heart center, we typically do not hold anticoagulation prior to cardiac catheterization. Here, we report our outcomes in a cohort of pediatric patients who underwent cardiac catheterization while on uninterrupted anticoagulation.

## Materials and Methods

This was a retrospective study performed with Institutional Review Board approval (protocol number CHLA-19-00456). Patients who were treated with systemic anticoagulation and underwent cardiac catheterization from 11/2014 to 10/2019 at our institution were identified using institutional databases and retrospectively reviewed. Exclusion criteria included interruption of systemic anticoagulation prior to cardiac catheterization and individuals on aspirin monotherapy.

Data collected included patient demographics, cardiac diagnosis, date of last cardiac surgery, indication for anticoagulation, type of anticoagulation used, whether arterial or venous access was utilized, largest sheath size, use of ultrasound for vascular access, interventions performed, preoperative and intraoperative labs, hemostasis method and time, and adverse events. For patients that received manual pressure for hemostasis, times were estimated by time stamps for sheath removal and dressing placement in the electronic medical record. Adverse events were classified as minor, moderate, major, or catastrophic according to previously published and tested definitions [13].

### Statistical Analysis

Continuous variables are presented as median [range] as they were not normally distributed. The statistical analysis was done with JMP Pro 15.1 (SAS institute Inc., Cary, NC) and Microsoft Excel 16.16.10.

## Results

One hundred fifty-one cardiac catheterizations were identified in 111 patients from 11/2014 to 10/2019 who were being treated with anticoagulation. Seven cases were excluded because anticoagulation was held prior to catheterization. The median age and weight of the remaining patients were 6.3 [0.01–20.9] years and 19.3 [2.1–172.5] kg, respectively (Table 1). Seventy-eight (54%) catheterizations involved inpatients. The most common cardiac diagnoses among the cohort were single ventricle (*n* = 41), conotruncal defects (*n* = 37), and structurally normal heart (*n* = 16) (Table 1).

**Table 1:** Baseline characteristics of cohort population

Patient characteristics	
Age (years)	6.3 [0.01–20.9]
Weight (kg)	19.3 [2.1–172.5]
Cardiac diagnoses	
Single ventricle	41 (28.5)
Conotruncal defects (i.e., TGA, TOF, DORV)	37 (25.7)
Normal heart	16 (11.1)
Cardiomyopathy/myocarditis	15 (10.4)
Pulmonary stenosis	10 (6.9)
Septal defects (i.e., ASD, VSD, or AV canal)	8 (5.6)
Coronary aneurysm	8 (5.6)
Primary mitral or tricuspid valve disease	5 (3.5)
Coarctation/arch hypoplasia	2 (1.4)
SVC stenosis	1 (0.7)
TAPVR	1 (0.7)
Cardiac surgery on same admission	28 (19.4)
Type of patient	
Inpatient	78 (54.2)
Outpatient	66 (45.8)
Type of anticoagulation (*n* = 144)	
Warfarin	57 (39.6)
Heparin	52 (36.1)
Enoxaparin	25 (17.4)
Fondaparinux	5 (3.5)
Warfarin and heparin	3 (2.1)
Rivaroxaban	2 (1.4)
On antiplatelet agent	53 (36.8)

Data shown as median [range] or n (%)

*ASD* atrial septal defect, *AV* atrioventricular, *DORV* double outlet right ventricle, *SVC* superior vena cava, *TAPVR* total anomalous pulmonary venous return, TGA transposition of the great arteries, *TOF* tetralogy of Fallot, *VSD* ventricular septal defect

### Indication for Anticoagulation and Anticoagulation Levels

The most common indications for anticoagulation were arterial or venous thrombosis (*n* = 45), Fontan physiology including primary prophylaxis, fenestration, or thrombus (*n* = 32), mechanical valve (AV valve *n* = 23, aortic valve *n* = 4), extracorporeal membrane oxygenation (ECMO, *n* = 16), heart failure (*n* = 9), and coronary artery aneurysm (*n* = 8) (Table 2). The anticoagulation medications used were warfarin (*n* = 57), heparin (*n* = 52), enoxaparin (*n* = 25), fondaparinux (*n* = 5), rivaroxaban (*n* = 2), and both heparin and warfarin (*n* = 3). Fifty-three patients were on antiplatelet agents in addition to systemic anticoagulation (Table 1). Pre-catheterization INR was available in 58 patients on warfarin and the median was 2.35 [1.4–6.6] (Table 3). Pre-catheterization anti-Xa was available in 43 patients on heparin infusion and 21 patients on enoxaparin; the medians were 0.41U/ml [0.05–1.96] and 0.63 [0.23–1], respectively**.**

**Table 2:** Indications for systemic anticoagulation

Indication for anticoagulation	*n* = 144
Systemic thrombus	45 (31.3)
Fontan (fenestration, primary prophylaxis, or thrombus)	32 (22.2)
Mechanical valve	27 (18.8)
ECMO	16 (11.1)
Heart failure	9 (6.3)
Coronary aneurysm	8 (5.5)
Other	3 (2.1)
Stent	2 (1.4)
Stroke	2 (1.4)

Data shown as n (%)

*ECMO* extracorporeal membrane oxygenation

**Table 3:** Procedural characteristics and anticoagulation levels

Procedural characteristics and anticoagulation levels	
Procedural length (min)	122.5 [15–760]
Intervention performed	96 (66.7)
Ultrasound used for vascular access	78 (54.2)
Vascular access type	
Both arterial and venous	95 (66.0)
Arterial only	8 (5.6)
Venous only	41 (28.5)
Arterial sheath size	
7 Fr	1 (0.7)
5 Fr	29 (20.1)
4 Fr	50 (34.7)
3.3 Fr	7 (4.9)
20 G catheter or micro-introducer kit dilator	16 (11.1)
Venous sheath size > 7Fr	32 (22.2)
Pre-catheterization anticoagulation levels	
INR while on warfarin (*n* = 58)	2.35 [1.4–6.6]
Anti-Xa while on heparin (*n* = 43)	0.41 [0.05–1.96]
Anti-Xa while on enoxaparin (*n* = 21)	0.63 [0.23–1]
Opening activated clotting time (sec) (*n* = 101)	184 [77–308]
Additional procedural anticoagulation administered	
Heparin	87 (60.4)
Bivalirudin	5 (3.5)
Hemostasis method	
Manual pressure	141 (97.9)
Figure “8” stitch	3 (2.1)
Time to hemostasis (min)*	18.0 [5–76]

Data shown as median [range] or n (%)

*The median time to hemostasis in all catheterizations using manual pressure

*Fr* french, *G* gauge, *INR* international normalized ratio

### Procedural Characteristics

Ultrasound was utilized for vascular access in 78 cases (54%) (Table 3). Both arterial and venous access were obtained in 95 cases (66%), only arterial access in 8 cases (6%), and only venous access in 41 cases (29%). The largest arterial access obtained was a 7 Fr sheath in 1 case. The most commonly used sheath sizes for arterial access were a 4 Fr sheath in 50 cases, 5 Fr sheath in 29 cases, 3.3 Fr sheath in 7 cases, and a 20 G arterial catheter or a dilator from a 4 Fr micro-introducer kit in 16 cases. The largest venous sheath was 22 Fr, and a sheath larger than 7 Fr was used in 32 cases. The opening activated clotting time was 184 [77–308] seconds (*n* = 101). Additional anticoagulation was administered during catheterization in 92 cases, including heparin in 87 cases and bivalirudin in 5 cases. The median bolus doses of heparin and bivalirudin given were 72 units/kg [17.5–101.9 units/kg] and 0.5 mg/kg [0.45–0.50 mg/kg)], respectively.

Interventions or special diagnostic procedures (endomyocardial or liver biopsy) (*n* = 159) were common and performed in 96 cases (67%) (Table 4). They included angioplasty (*n* = 67), stent implantation (*n* = 31), vascular embolization (*n* = 18), transeptal puncture (*n* = 10), transjugular liver biopsy (*n* = 10), thrombectomy (*n* = 11), Fontan fenestration occlusion (*n* = 6), endomyocardial biopsy (*n* = 3), valvuloplasty (*n* = 2), and balloon atrial septostomy (*n* = 1). The median length of the procedure was 122.5 [15–760] minutes, and the median time to achieve hemostasis for cases that received manual pressure was 18.0 [range: 5–76, IQR: 13–25] minutes.

**Table 4:** Interventions performed during 96 CC

Interventions/special diagnostic procedures	*n* = 159
Angioplasty	67 (42.1)
Stent implantation	31 (19.5)
Vascular embolization	18 (11.3)
Thrombectomy	11 (6.9)
Transeptal puncture	10 (6.3)
Liver biopsy	10 (6.3)
Fontan fenestration occlusion	6 (3.8)
Endomyocardial biopsy	3 (1.9)
Valvuloplasty	2 (1.3)
Balloon atrial septostomy	1 (0.6)

Data shown as n (%)

### Adverse Events and Hematologic Complications

Adverse events were present in 11 cases (7.6%). There were six minor (4.2%) events (transient arrhythmia in three, bleeding from access site on the day following catheterization, small hematoma, and sheath dislodgement). There was one moderate (0.7%) event (non-occlusive arterial thrombus). There were four major (2.8%) events (ventricular fibrillation, transient complete heart block, and respiratory arrest in two). Bleeding complications were present in two cases (1.4%), both of which were classified as minor. Both patients were on warfarin as the anticoagulation agent with INRs of 2.8 and 3.1.

## Discussion

This is the first report specifically describing outcomes of pediatric patients who underwent cardiac catheterization while on uninterrupted anticoagulation. Our single-center data suggest that this practice is safe and does not substantially increase the risk of bleeding complications in a cohort of patients that varied in age, size, diagnosis, and type of intervention performed. The most common indications for anticoagulation were venous or arterial thrombosis, Fontan physiology, and mechanical valve thrombus prophylaxis. Patients were most likely to be on warfarin (39.6%) or heparin (36.1%) for anticoagulation.

Patients on warfarin therapy for a mechanical valve are most likely to benefit from this practice, as interruption of warfarin therapy is not ideal. The ability to continue warfarin therapy avoids the need for bridging, a period without anticoagulation therapy, as well as the need to transition the patient back to warfarin therapy. This practice can help to minimize the number of lab draws and length of hospital stay, particularly in young infants where management of warfarin can be challenging. In adults this has been shown to be a safer practice in patients undergoing pacemaker or defibrillator implantation with lower rates of device-pocket hematoma in the group that did not have interruption of warfarin therapy [14]. Furthermore, it has also been shown to be safe in adults undergoing diagnostic cardiac catheterization via the femoral or radial arteries and coronary interventions via the radial artery [6].

### Hemostasis

One of the outcome measures of this study was time to hemostasis. The median time to hemostasis in all catheterizations using manual pressure was 18.0 [range: 5–76, IQR: 13–25] minutes, with 62% requiring more than 15 min of compression. Waragai et al. published a study assessing hemostasis times in pediatric patients, which demonstrated a mean time to hemostasis of 16.1 min, with 36% requiring more than 15 min of compression [15]. Our cohort did not differ substantially from this. Data in adults support this finding as well. Lippe et al. demonstrated that hemostasis times are similar in adults undergoing radial artery catheterization whether or not they were on uninterrupted warfarin [16].

### Complications

Overall complication rates (7.6%) seen in this study are consistent with previously published rates which have ranged from 7.3 to 15% [10, 11, 17]. When specifically analyzing bleeding complications our cohort had a similar rate (1.4%) of complications when compared to Mehta et al. (0.7%) and Vitiello et al. (0.8%) [10, 11]. None of the bleeding complications in our cohort were classified as major compared to the 4.1% and 11.9% classified as such by Mehta et al. and Vitiello et al., respectively. In comparing our cohort to the aforementioned published cohorts, it is important to note that our patients were, on average, suffering from more severe illness with over half requiring inpatient care and over 10% requiring ECMO.

While we were able to show the complications are relatively minor and rare while performing a variety of transcatheter interventions, it is important to tailor the anticoagulation plan to the patient. The diagnosis, indication for anticoagulation, bleeding risk of the planned intervention, access site, and anticipate sheath sizes should all be considered. Additionally, it is important to assess the activated clotting time and modify the intraprocedural anticoagulation dose if necessary [18]. In our population we either omitted or modified the intraprocedural anticoagulation dose. Another procedural modification that can be considered is use of a suture-mediated closure device, particularly in obese patients in whom bleeding or hematoma development may be more difficult to immediately recognize.

### Limitations

There were several limitations to our study. This is a retrospective study with data from a single center with a relatively small sample size. The patient cohort is heterogenous with a variety of diagnoses, indications for anticoagulation, anticoagulation therapies, and therapeutic goals. Additionally, there was no control group to compare the cohort’s outcomes to. Despite these limitations, there are several important observations that can be made from this study.

## Conclusion

This study demonstrates cardiac catheterization with a variety of interventions and utilizing a range of arterial and venous sheaths may be performed safely in pediatric patients on uninterrupted anticoagulation. Bleeding-related complications were similar to previously reported rates in patients not on anticoagulation, and observed complications were all mild in nature. Further studies in a larger cohort of patients are needed to better characterize the effects of uninterrupted vs interrupted anticoagulation in pediatric patients undergoing cardiac catheterization.

## References

[1] Douketis JD, Spyropoulos AC, Spencer FA, Mayr M, Jaffer AK, Eckman MH (2012). Perioperative management of antithrombotic therapy. Antithrombotic therapy and prevention of thrombosis: American college of chest physicians evidence-based clinical practice guidelines. Chest.

[2] Levine MN, Raskob G, Landefeld S, Kearon C (2001). Hemorrhagic complications of anticoagulant treatment. Chest.

[3] Naidu SS, Abbott JD, Bagai J, Blankenship J, Garcia S, Iqbal SN (2021). SCAI expert consensus update on best practices in the cardiac catheterization laboratory. Catheter Cardiovasc Interv.

[4] Lip GYH, Windecker S, Huber K, Kirchhof P, Marin F, Berg JMT (2014). Management of antithrombotic therapy in atrial fibrillation patients presenting with acute coronary syndrome and/or undergoing percutaneous coronary or valve interventions: a joint consensus document of the European Society Of Cardiology Working Group on. Eur Heart J.

[5] Kowalewski M, Suwalski P, Raffa GM, Słomka A, Kowalkowska ME, Szwed KA (2016). Meta-analysis of uninterrupted as compared to interrupted oral anticoagulation with or without bridging in patients undergoing coronary angiography with or without percutaneous coronary intervention. Int J Cardiol.

[6] Baker NC, O’Connell EW, Htun WW, Sun H, Green SM, Skelding KA (2014). Safety of coronary angiography and percutaneous coronary intervention via the radial versus femoral route in patients on uninterrupted oral anticoagulation with warfarin. Am Heart J.

[7] Birnie DH, Healey JS, Wells GA, Ayala-Paredes F, Coutu B, Sumner GL (2018). Continued vs. interrupted direct oral anticoagulants at the time of device surgery, in patients with moderate to high risk of arterial thrombo-embolic events (BRUISE CONTROL-2). Eur Heart J..

[8] Cappato R, Marchlinski FE, Hohnloser SH, Naccarelli GV, Xiang J, Wilber DJ (2015). Uninterrupted rivaroxaban vs uninterrupted vitamin K antagonists for catheter ablation in non-valvular atrial fibrillation. Eur Heart J.

[9] Melillo E, Carbone A, Rago A, Papa AA, Onofrio DA, Nigro G (2020). Update on direct oral anticoagulants in atrial fibrillation patients undergoing cardiac interventional procedures: from clinical trials to real-world evidence. J. Cardiovasc Pharmacol.

[10] Mehta R, Lee KJ, Chaturvedi R, Benson L (2008). Complications of pediatric cardiac catheterization: a review in the current era. Catheter Cardiovasc Interv.

[11] Vitiello R, Mccrindle BW, Nykanen D, Freedom RM, Benson LN (1998). Complications associated with pediatric cardiac catheterization. J Am Coll Cardiol.

[12] Taggart NW, Gordon BM, Morgan GJ, Goldstein BH (2019). Variation in Anticoagulation Practices in the Congenital Cardiac Catheterization Lab: Results of a Multinational PICES Survey. Pediatr Cardiol.

[13] Bergersen L, Gauvreau K, Jenkins KJ, Lock JE (2008). Adverse event rates in congenital cardiac catheterization: a new understanding of risks. Congenit Heart Dis.

[14] Birnie DH, Healey JS, Wells GA, Verma A, Tang AS, Krahn AD (2013). Pacemaker or defibrillator surgery without interruption of anticoagulation. N Engl J Med..

[15] Waragai T, Morgan G, Ralston T, Chaturvedi R, Lee KJ, Benson L (2011). Vascular hemostasis bandage compared to standard manual compression after cardiac catheterization in children. Catheter Cardiovasc Interv.

[16] Lippe CM, Reineck EA, Kunselman AR, Gilchrist IC (2015). Warfarin: impact on hemostasis after radial catheterization. Catheter Cardiovasc Interv.

[17] Bergersen L, Marshall A, Gauvreau K, Beekman R, Hirsch R, Foerster S (2010). Adverse event rates in congenital cardiac catheterization—A multi-center experience. Catheter Cardiovasc Interv.

[18] Hamam I, Daoud EG, Zhang J, Kalbfleisch SJ, Augostini R, Winner M (2013). Impact of international normalized ratio and activated clotting time on unfractionated heparin dosing during ablation of atrial fibrillation. Circ Arrhythmia Electrophysiol.

